# The structure of tubulin-binding cofactor A from *Leishmania major* infers a mode of association during the early stages of microtubule assembly

**DOI:** 10.1107/S2053230X15000990

**Published:** 2015-04-21

**Authors:** Keri L. Barrack, Paul K. Fyfe, William N. Hunter

**Affiliations:** a Division of Biological Chemistry and Drug Discovery, College of Life Sciences, University of Dundee, Dow Street, Dundee DD1 5EH, Scotland

**Keywords:** chaperone, helical fold, protein–protein interactions, selenomethionine, tubulin-binding protein

## Abstract

Tubulin-binding cofactor A (TBCA) participates in microtubule formation, a key process in eukaryotic biology to create the cytoskeleton. There is little information on how TBCA might interact with β-tubulin en route to microtubule biogenesis. To address this, the protozoan *Leishmania major* was targeted as a model system. The crystal structure of TBCA and comparisons with three orthologous proteins are presented. The presence of conserved features infers that electrostatic interactions that are likely to involve the C-terminal tail of β-tubulin are key to association. This study provides a reagent and template to support further work in this area.

## Abstract 2

The structure of a tubulin-binding cofactor from *L. major* is reported and compared with yeast, plant and human orthologues.

## 1. Introduction   

Protozoan parasites are the causal agents of serious infections that affect humans and livestock. Research to improve knowledge of parasite biology has been driven by the need to know the enemy and to inform on strategies for treatment and prevention (Hunter, 2009 ▶). It has also become evident that some protozoan pathogens, as primitive eukaryotes, provide excellent model systems to support basic research. In this respect, the trypanosomatid parasites have proven particularly valuable (Cross, 2005 ▶).

We are exploiting trypanosomatids as a model system to help dissect the contributions that a class of proteins termed tubulin-binding cofactors and ancillary proteins make to the complex process whereby tubulin is assembled into microtubules (Fleming *et al.*, 2010 ▶, 2013 ▶). Tubulin polymerization is key to formation of the eukaryotic cytoskeleton (Lundin *et al.*, 2010 ▶). The process involves first the supply of correctly folded α- and β-tubulin forms, which form heterodimers, followed by the correct assembly of the microtubule polymer whilst avoiding aggregation. Post-translational modifications of the tubulin subunits also occur and must be carefully regulated. Tubulin-binding cofactor (TBC) proteins (Lopez-Fanarraga *et al.*, 2001 ▶), of which there are at least five, are intimately involved in this assembly process, but the details about specific contributions are limited. Microtubules are dynamic structures that undergo both assembly and disassociation, and it can be supposed that accessory proteins will play a role in both processes. In order to underpin dissection of TBC function and delineation of structure–activity relationships, we have instigated studies of the trypanosomatid proteins and now report a crystallographic study of one, TBCA, from *Leishmania major*. This protein is thought to make an early contribution to the assembly process through delivery of β-tubulin onto a second protein, TBCD. In one model for microtubule biogenesis this TBCD–β-tubulin complex then facilitates α/β-tubulin heterodimer formation (Lundin *et al.*, 2010 ▶). We describe the high-resolution crystallographic analysis of *L. major* TBCA (*Lm*TBCA) and provide comparisons with orthologous structures derived from yeast, plant and mammalian sources. Conserved features suggest several structural factors that might be important for interactions between TBCA and a cognate tubulin and provide a model to help formulate hypotheses and further dissect a key aspect of eukaryotic biology.

## 2. Materials and methods   

### 2.1. Cloning, expression and purification of recombinant protein   

The gene predicted to encode TBCA from *L. major* Friedlin in GeneDB (LmjF.32.2970; Logan-Klumpler *et al.*, 2012 ▶) was amplified by PCR from genomic DNA and subcloned into the expression vector pET-15b-TEV (modified from pET-15b, Novagen). Under the control of the T7 promoter, this vector produces protein with an N-terminal hexahistidine tag that is cleavable by tobacco etch virus (TEV) protease. The resulting plasmid was transformed into *Escherichia coli* strains BL21 (DE3) Gold and B834 (DE3) for the production of native and selenomethionine (SeMet)-derived proteins, respectively. Typically, bacteria were cultured at 310 K to an optical density at 600 nm of 0.5–0.6 in Luria–Bertani medium containing 50 mg l^−1^ carbenicillin or in methionine-deficient medium supplemented with 50 mg l^−1^ carbenicillin and 40 mg l^−1^ selenomethionine (Molecular Dimensions). Protein production was then induced with 1 m*M* isopropyl β-d-1-thiogalactopyranoside and growth continued for approximately 16 h at 295 K prior to harvesting the cells by centrifugation (3500*g* for 30 min at 277 K). The cell mass was stored frozen at 253 K until required.

The cells were thawed on ice and then lysed using a French press at 110 MPa, and the lysate was clarified by centrifugation at 37 500*g* for 30 min at 277 K. The soluble supernatant was filtered (0.2 µm) and loaded onto a 5 ml HisTrap HP column (GE Healthcare) for an initial affinity-chromatography capture step. The product was then eluted between 150 and 180 m*M* imidazole; fractions containing *Lm*TBCA were pooled and treated with TEV protease at 303 K for 2 h. Dialysis at room temperature to remove excess imidazole was followed by reverse affinity chromatography prior to a final purification step by size-exclusion chromatography using a calibrated Superdex 75 26/60 gel-filtration column in 50 m*M* Tris–HCl, 250 m*M* NaCl pH 7.5. In all purifications that were carried out, the gel-filtration trace of *Lm*TBCA indicated the presence of two species in an approximate ratio of 2.5:1 with approximate molecular weights of 27 and 43 kDa, respectively. The theoretical molecular weight is about 14 kDa. The protein from each peak was pooled separately and both samples were buffer-exchanged into 10 m*M* Tris–HCl, 100 m*M* NaCl pH 7.5 and concentrated using centrifugal concentrators (Sartorius) prior to crystallization trials. *Lm*TBCC lacks tryptophan, so chromatograms were obtained at both 215 and 280 nm and the protein concentration was determined by measurement of the absorbance at 595 nm using Coomassie (Bradford) protein-assay reagent (Thermo Fisher Scientific) with bovine serum albumin as a reference. Protein mass was verified by matrix-assisted laser desorption/ionization time-of-flight mass spectrometry (MALDI-TOF; University of Dundee Proteomics Facility) and sample purity was assessed by SDS–PAGE.

### 2.2. Crystallization   

The lower abundance, higher molecular-weight sample did not produce any crystals. The other sample of full-length *Lm*TBCA gave highly ordered crystals in hanging-drop vapour-diffusion experiments when a protein solution at 4 mg ml^−1^ in 50 m*M* Tris–HCl pH 7.5, 100 m*M* NaCl was mixed with an equal volume of reservoir solution [0.2 *M* (NH_4_)_2_HPO_4_, 1.6 *M* ammonium sulfate]. Crystals with typical dimensions of 1.0 × 0.2 × 0.2 mm appeared within 1–3 d at 291 K. Isomorphous (Table 1 ▶) but much smaller SeMet-*Lm*TBCA crystals were grown using the same approach with the protein at a concentration of 3.8 mg ml^−1^ and using a reservoir solution consisting of 0.3 *M* LiCl, 1.0 *M* ammonium sulfate.

### 2.3. X-ray data collection and processing, structure determination and refinement   

Crystals were cryoprotected in mother liquor supplemented with 25%(*v*/*v*) glycerol and were then flash-cooled in liquid nitrogen before in-house screening and characterization using a Rigaku MicroMax-007 HF rotating-anode source equipped with a Saturn 944 HG+ CCD detector with the sample maintained at 100 K. Diffraction data were subsequently collected from both native and SeMet-derivative crystals on beamline ID23-2 at the European Synchrotron Radiation Facility (ESRF) using a MAR Mosaic 225 CCD detector. This beamline has a fixed wavelength of 0.8726 Å, which is a higher energy than that of the optimum selenium *f*′′ edge, but still sufficient to give values for *f*′ and *f*′′ of approximately −1.2 and 3.0, respectively. The anomalous dispersion signal was judged to be within the range that can provide useful phase information (Micossi *et al.*, 2002 ▶).

Phases were determined experimentally by single-wavelength anomalous dispersion (SAD) methods using the SeMet derivative. Data to 2.3 Å resolution were integrated using *XDS* (Kabsch, 2010 ▶) and then processed with *POINTLESS* and *SCALA* (Evans, 2006 ▶; Evans & Murshudov, 2013 ▶). Initial phases were generated based on four selenium positions using *AutoSol* in *PHENIX* (Adams *et al.*, 2010 ▶). The resulting electron-density map (figure of merit 0.43) was of excellent quality and allowed the first model of 89 residues to be built. At this stage the *R*_work_ and *R*_free_ values were 29 and 33%, respectively, and the map–model correlation coefficient was 0.74.

The isomorphous native crystals diffracted to higher resolution (1.9 Å) and data from one of them were processed by combining *MOSFLM* (Battye *et al.*, 2011 ▶) with *POINTLESS* and *SCALA*. Molecular replacement and rigid-body refinement (*Phaser*; McCoy *et al.*, 2007 ▶) initiated refinement using this higher resolution data. The programs *Coot* (Emsley *et al.*, 2010 ▶) and *REFMAC*5 (Murshudov *et al.*, 2011 ▶) were used in turn to carry out several rounds of electron-density and difference density map inspection, model manipulation and incorporation of solvent and a sulfate and a glycerol in combination with refinement calculations. *MolProbity* (Chen *et al.*, 2010 ▶) was used to inspect model geometry in combination with the validation tools provided in *Coot*. Crystallo­graphic statistics are presented in Table 1 ▶.

## 3. Results and discussion   

### 3.1. Structural overview   

The structure of *Lm*TBCA was determined by the SAD approach and was refined to 1.9 Å resolution. Although the structures of orthologues were known (see below) attempts to solve the structure by molecular replacement failed, hence the recourse to experimental phases targeting a selenomethionine derivative.

**Figure 1:**
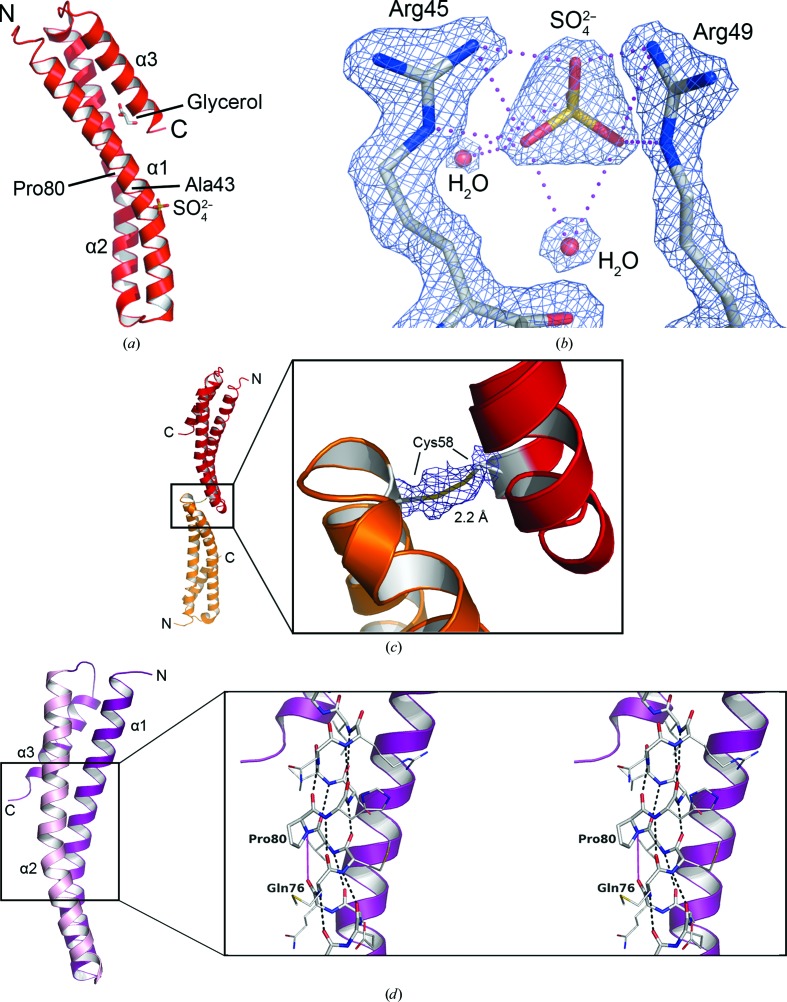
(*a*) Overall structure of *Lm*TBCA. The helices are represented as red ribbons. Ala43 and Pro80 are labelled as the approximate positions of bends in α1 and α2. A sulfate is bound to the surface of α1. (*b*) A closer view of the anion interacting with Arg45 and Arg49 is shown as gold (S) and red (O) sticks. The arginine residues are coloured by element (C, grey; N, blue) and two water molecules are depicted as red spheres. Blue mesh represents electron density (2*mF*_o_ − *DF*_c_ contoured at 2σ) and magenta dotted lines indicate potential hydrogen bonds. (*c*) The *Lm*TBCA monomer (red) and a symmetry-related molecule (orange) are linked by a disulfide bond between Cys58 residues with side-chain atoms shown as sticks (C, grey; S, gold). The OMIT *F*_o_ − *F*_c_ density for the side-chain atoms of Cys58 and the symmetry mate is depicted as chickenwire and contoured at 2σ. (*d*) Backbone hydrogen bonds on α2. The expanded area is a stereoview of α1 and α3 as purple ribbons. α2 is depicted as pale pink ribbons (left) or as sticks coloured by element (right). Black dashed lines represent standard α-helical hydrogen bonds. Pro80 disrupts this bonding pattern. The magenta line between Pro80 N and Gln76 O represents a distance of 4.20 Å.

The asymmetric unit contains a single polypeptide. There was no reliable electron density for the first 18 residues and the model comprises residues Glu19–Ser125. The protein fold consists of three α-helices (α1–α3) aligned in an antiparallel fashion. Helices α1–α2 and α2–α3 are linked by two short segments. The molecule is approximately 59 Å in length and about 23 Å at the widest point (Fig. 1 ▶ *a*). Helices α1 and α2, each comprising ten helical turns, are distorted, with bends of approximately 20–25°, giving the molecule an overall curved appearance. They are slightly twisted with respect to each other, and α3, with only four helical turns, is tightly incorporated into the bundle, interacting with the N-terminal and C-terminal regions of α1 and α2, respectively.

The crystals were grown in the presence of ammonium sulfate and a well ordered sulfate was observed to interact with Arg45 and Arg49 in the proximity of the distortions noted in α1 and α2 (Figs. 1 ▶ *a* and 1 ▶ *b*). The occupancy of this anion-binding site is explained by the crystallization conditions, but it may be indicative of a biologically relevant interaction site where a negatively charged or polar entity may bind. A molecule of glycerol, which was used as the cryoprotectant, is also bound to the protein, linking Glu41 on α1 and Asp119 on α3 (not shown).

Although a single polypeptide constitutes the asymmetric unit, there is a disulfide linkage between Cys58 at the C-terminal end of α1 and the corresponding residue of the neighbouring molecule related by the symmetry operation −*x*, −*x* + *y*, −*z* + 1/3 (Fig. 1 ▶ *c*). These two polypeptide chains are related by a crystallographic twofold axis of symmetry, which is parallel to the *b* unit-cell edge. *Lm*TBCA is therefore able to form a covalent dimer. The S atoms are 2.2 Å apart in well defined electron density (Fig. 1 ▶ *c*). The residues immediately adjacent to Cys58 display higher *B* factors compared with the rest of the molecule. The mean *B* factor of residues Asp55–Pro66 is 56.0 Å^2^ *versus* 27.3 Å^2^ for the protein as a whole, suggesting a degree of flexibility at Cys58 near the α1–α2 loop. However, the formation of this Cys58–Cys58 disulfide bond may have stabilized the structure of the α1–α2 loop, and as Cys58 is not a conserved residue in TBCA sequences, it may have fortuitously aided in the crystallization of *Lm*TBCA. The lack of conservation would suggest that Cys58 may not have an important functional role. The solutions used during purification and crystallization were not supplemented with reducing agents and therefore it is likely that the covalent dimer is the species with a molecular weight of approximately 27 kDa observed in the size-exclusion gel-filtration traces. MALDI-TOF spectrometry of the sample that was used for crystallization also indicated the presence of a species with mass 28 423 Da that would correspond to the covalent dimer.

The helices generally present the standard 4_13_ hydrogen-bonding pattern and interhelical hydrogen-bonding inter­actions serve to align the helices with respect to each other. The bend in α2 mentioned above may be owing to the presence of Pro80, which disrupts the standard α-helix organization (Fig. 1 ▶ *d*). A number of hydrogen bonds close to Pro80 appear to be stretched. For example, the distance from the amide of Val79 to the carbonyl of Ala75 is 3.5 Å, while other neighbouring hydrogen bonds are between approximately 2.9 and 3.2 Å. The resolution of the structure does not allow us to be certain whether a real weakening of some hydrogen bonds occurs, but we judge it worth a comment. Interactions that link α1 and α2 are distributed along the length of the molecule; these include salt bridges formed by Lys29–Asp92 and Asp39–Arg85 pairings and a hydrogen bond between Glu50 and Gln68 (not shown).

### 3.2. A comparison with three orthologues   

**Figure 2:**
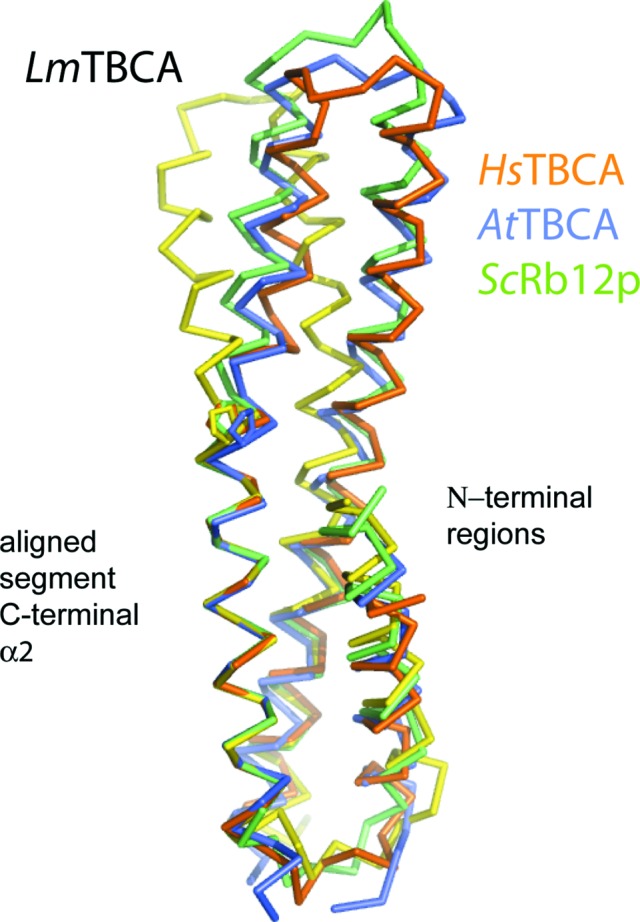
Superposition of four orthologous TBCA structures. The C^α^ atoms in the C-terminal segment of α2 were used for the superposition. The models and details of the least-squares superposition are *Lm*TBCA (yellow; superimposed residues 83–98; PDB entry 4cqi), *Hs*TBCA (orange; residues 68–83; PDB entry 1h7c), *Sc*Rbl2p (green; residues 66–81; PDB entry 1qsd) and *At*TBCA (blue; residues 63–82; PDB entry 3mxz).

The crystal structures of three TBCA orthologues have previously been reported. These are from the plant *Arabidopsis thaliana* (*At*TBCA; PDB entry 3mxz; Lu *et al.*, 2010 ▶), from *Homo sapiens* (*Hs*TBCA; PDB entry 1h7c; Guasch *et al.*, 2002 ▶) and from the yeast *Saccharomyces cerevisiae* (*Sc*Rbl2p; PDB entry 1qsd; Steinbacher, 1999 ▶). All three proteins display the same overall structure as *Lm*TBCA; that is, a bundle of three helices connected by short loops. However, the curvatures of the molecules differ (Fig. 2 ▶). The proline at position 80 in *Lm*TBCA is strictly conserved in these structures as shown below, and appears to contribute to a bend in α2 in all cases, although the effect appears most pronounced in the *Leishmania* protein. *Hs*TBCA has a different conformation, with α2 kinked in the opposite direction to that seen in *Lm*TBCA. Of the published models, *At*TBCA is most similar to *Lm*TBCA, with a root-mean-square deviation (r.m.s.d.) of 1.8 Å when 92 C^α^ atoms are aligned. Sequence similarity is also highest at about 31%, compared with 23 and 21% identity with *Sc*Rbl2p and *Hs*TBCA.

**Figure 3:**
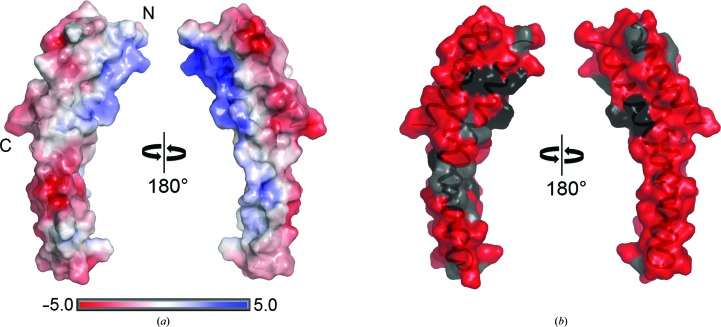
(*a*) Electrostatic potential of *Lm*TBCA. A van der Waals surface representation of *Lm*TBCA is coloured according to electrostatic protein contact potential (from −5*kT* e^−1^ in red to 5*kT* e^−1^ in blue) created using the programs *PDB*2*PQR* (Dolinsky *et al.*, 2004 ▶) and *APBS* (Baker *et al.*, 2001 ▶). (*b*) A van der Waals surface of *Lm*TBCA coloured by homology to related structures (*Hs*TBCA, *Sc*Rbl2p and *At*TBCA). Amino acids that are only present in *Lm*TBCA are coloured red. Increasing similarity is represented by a darkening greyscale with residues identical in all four species shown in black.

**Figure 4:**
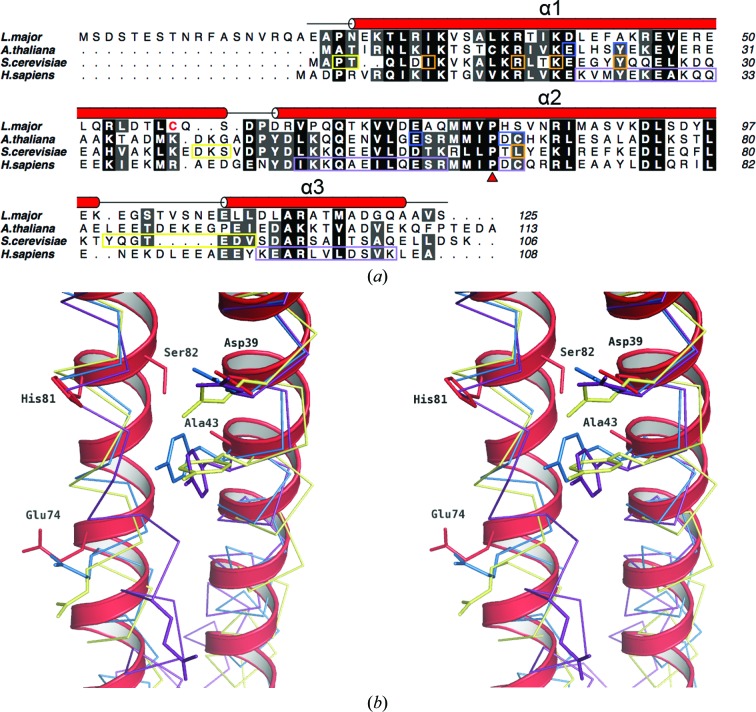
(*a*) Structure-based sequence alignment of *Lm*TBCA and the published structures of *At*TBCA, *Sc*Rbl2p and *Hs*TBCA. Residues highlighted in coloured boxes (blue, yellow and purple) are implicated in binding β-tubulin. Amino acids in orange boxes are thought to affect β-tubulin binding but are also located at the *Sc*Rbl2p homodimer interface. Cys58 is shown in red text and the location of the conserved proline (Pro80) is marked with a red triangle. Sequences were aligned with *MUSCLE* (Edgar, 2004 ▶) and the figure was prepared using *ALINE* (Bond & Schüttelkopf, 2009 ▶). (*b*) Stereo-image of *Lm*TBCA helices α1 and α2 (red ribbon) with C^α^ backbone traces of *At*TBCA (blue), *Sc*Rbl2p (yellow) and *Hs*TBCA (purple). Selected residues are shown as sticks of the same colours labelled according to *Lm*TBCA sequence and numbering. Residues at the corresponding positions of Asp39, Ala43 and Glu74 in *Lm*TBCA appear to be critical for β-tubulin binding in *A. thaliana*. His81 and Ser82 are also thought to play a functional role (see text). Structural alignments were calculated using secondary-structure matching (Krissinel & Henrick, 2004 ▶).

*Lm*TBCA α1 and α2 each possess an abundance of hydrophobic residues with side chains mainly directed towards the center of the trihelical bundle and providing a core to the fold. Electrostatic potential mapping indicates areas of localized polarity distributed over the protein surface. The concave exterior, when represented as a van der Waals surface, is mainly positive in charge in comparison with the rest of the molecule (Fig. 3 ▶ *a*). The overall calculated pI is 5.2 (*ProtParam*; Gasteiger *et al.*, 2005 ▶). The concentration of positive charge is in part attributed to conserved arginine and lysine residues on α1 (Fig. 3 ▶ *b*). The distribution of amino acids along α1 reveals that the majority of basic residues are localized on the surface just described (Fig. 4 ▶ *a*). The binding partner of TBCA, β-tubulin, is a highly conserved polypeptide (Sullivan & Cleveland, 1986 ▶) and is acidic, with a calculated pI of 4.6–4.7 (*ProtParam*). In addition, both α- and β-tubulin C-terminal segments are highly negatively charged and known to bind to microtubule-associated proteins and other cationic molecules (Cross *et al.*, 1991 ▶; Lefèvre *et al.*, 2011 ▶). The concave surface of *Lm*TBCA might therefore present a favourable site for interaction, driven by electrostatic attraction, with β-tubulin.

*Lm*TBCA lacks a significant hydrophobic surface site, which is a characteristic of proteins involved in interactions with unfolded or partially folded partners. An example would be the surfaces observed on GroEL of the archetypal bacterial protein-folding system (Fenton *et al.*, 1994 ▶) and also on members of the Hsp70 (heat-shock protein of 70 kDa mass) molecular chaperone family (Flynn *et al.*, 1991 ▶). This is in agreement with the hypothesis that β-tubulin is already in a folded state when initially presented to the tubulin-binding cofactors. Indeed, it has also been shown that TBCA does not recognize denatured β-tubulin (Archer *et al.*, 1998 ▶), so it would appear that the cofactors do not actually contribute to tubulin folding. What then is their function?

Tubulin, as mentioned, is a highly conserved molecule and an alignment of β-tubulin sequences from the same organisms as those for which TBCA structures have been produced indicates that between 70% (*S. cerevisiae* β-tubulin) and 85% (*H. sapiens* and *A. thaliana* β-tubulin) of residues are identical to *L. major* β-tubulin. This high level of similarity in a protein from a protozoan, a yeast, a plant and a mammal infers that the location of binding events involving β-tubulin are likely to be conserved. Interactions with the globular surface might then require some conserved feature on the partner molecules. Since the overall sequence identity between TBCAs is much lower than that of the β-tubulins, localized regions of high conservation offer the greatest interaction potential. β-Tubulin sequences diverge mainly at the C-terminal tail, a site that has been implicated in many other microtubule-related interactions (see, for example, Cross *et al.*, 1991 ▶; Lefèvre *et al.*, 2011 ▶) and, without detailed structural knowledge, cannot be ruled out as the site of interaction with TBCA.

Different theories of how TBCA interacts with β-tubulin have been presented. Peptide-mapping and competition experiments suggest that β-tubulin interacts with all three helices of *Hs*TBCA (Guasch *et al.*, 2002 ▶). α3 was not considered to be essential, but binding activity was diminished when it was removed. Two specific amino-acid mutations, D66E and C67S, appeared to influence binding. These residues are not strictly conserved in this protein family; for example, Asp66 and Cys67 in *Hs*TBCA correspond to His81 and Ser82 in *Lm*TBCA. Nevertheless, that mutations have been shown to have an effect on function suggests a contribution to the binding event. Immediately preceding His81 and Ser82 in *Lm*TBCA is the highly conserved Pro80 that may contribute to the distortion of α2 observed in TBCA structures. Although the binding data and crystallographic models suggest that Pro80, and the corresponding residue in orthologues, is not directly involved in contacts with β-tubulin, the strict conservation suggests a contribution to the overall shape and structure of TBCA.

Mutagenesis and co-immunoprecipitation studies with *At*TBCA revealed that Glu20, Tyr24 and Glu57 were critical for binding β-tubulin (Lu *et al.*, 2010 ▶). Mutation of each of these individually to alanine in *At*TBCA resulted in the absence of a TBCA–β-tubulin complex. According to sequence and structural alignments, all three residues are strictly conserved in *Hs*TBCA and in *Sc*Rbl2p; only Glu57 differs, being replaced conservatively by Asp57. These three positions correspond to Asp39, Ala43 and Glu74 in *Lm*TBCA. The residues are located in the C-terminal half of α1 and the N-terminal half of α2, which are the regions of greatest variation between these four proteins in superpositions using secondary-structure matching procedures (*SSM*; Krissinel & Henrick, 2004 ▶). If a plane were to dissect the protein in half, approximately at the level of the C-terminus in Fig. 1 ▶(*a*), the mean C^α^ deviations are 0.8 Å greater in the lower portion of the molecule than in the upper part. The negatively charged side-chain atoms of Asp39 and Glu74 are exposed on the surface of the helix bundle (Fig. 4 ▶ *b*). The hydrophobic Ala43, however, is more buried and the C^β^ atom extends towards α2, unlike the large side group of Tyr24 in *At*TBCA, which projects out towards the solvent on the same surface as Glu20. The ability of a single Tyr-to-Ala mutation to eliminate binding suggests a critical functional role, but this is contradicted by the presence of an alanine in native *Lm*TBCA. Perhaps *Lm*TBCA displays lower affinity for β-tubulin as a result or there may be additional or alternative contributions made by amino acids elsewhere. For example, the distinct curvature of *Lm*TBCA could present residues along the entire length of the helices, including the positively charged region of α1 discussed, towards the binding partner.

Evidence against the potential binding pattern just described is provided by computational docking calculations, which suggest that the homodimeric *Sc*Rbl2p interacts with β-tubulin *via* the short loops rather than the helices (You *et al.*, 2004 ▶). In this case, α1 and α2 form a dimer interface and a number of the residues discussed above make contacts with or are buried by the second molecule (Steinbacher, 1999 ▶). However, of the known TBCA structures, only *Sc*Rbl2p is reported to form such a homodimeric assembly. Although the dimer interface is notably hydrophilic (Steinbacher, 1999 ▶), the arrangement of the few hydrophobic residues forming intermolecular contacts is absent in *Lm*TBCA, *At*TBCA and *Hs*TBCA. Although *Lm*TBCA did display a dimeric assembly, it is distinct from that observed for ScRbl2p and is driven by the formation of a disulfide linkage. Other intermolecular contacts in the crystal lattice are distributed around the surface and are not clustered to resemble a biologically relevant dimerization interface.

## 4. Concluding remarks   

A bacterial recombinant protein-production system for *Lm*TBCA has been prepared, the protein has been purified and the structure determined at high resolution using a SAD approach applied to a SeMet derivative. A helical bundle structure is described and comparisons with orthologous proteins have been carried out. We do not ascribe any biological significance to the observation of a covalent dimer of *Lm*TBCA formed owing to a disulfide linkage. A conserved proline appears to cause a distortion of α1 and overall the molecule has a curved shape. The presence of a localized region enriched in basic residues, which are conserved in TBCA from evolutionarily diverse species, together with the overall acidic properties of the C-terminal tails of β-tubulins, hints that electrostatic forces might be relevant for complex formation, but further work is required to address such a hypothesis.

## Supplementary Material

PDB reference: tubulin-binding cofactor A, 4cqi

## Figures and Tables

**Table 1:** Crystallographic statistics Values in parentheses are for the highest resolution shell.

	SeMet *Lm*TBCA	Native *Lm*TBCA
Resolution range ()	39.02.3 (2.42.3)	39.01.9 (2.01.9)
Space group	*P*3_1_21	*P*3_1_21
Unit-cell parameters (, )	*a* = *b* = 76.8, *c* = 39.5, = = 90, = 120	*a* = *b* = 76.8, *c* = 39.4, = = 90, = 120
Wavelength ()	0.8726	0.8726
No. of reflections	73555 (10683)	130402 (19204)
No. of unique reflections	6205 (878)	10821 (1549)
*R*_merge_ † (%)	13.8 (61.0)	7.3 (44.8)
*R*_p.i.m._ ‡ (%)	5.9 (26.4)	2.2 (13.2)
Completeness (%)	100 (100)	100 (100)
*I*/(*I*)	19.5 (6.0)	24.9 (6.8)
Multiplicity	11.9 (12.2)	12.1 (12.4)
Wilson *B* factor (^2^)	35.3	20.9
*R*_work_ § (%)		18.5
*R*_free_ ¶ (%)		22.7
R.m.s.d., bonds ()		0.0134
R.m.s.d., angles ()		0.1586
Total protein residues		107
Total protein atoms		855
No. of solvent atoms		90
No. of sulfates		1
No. of glycerols		1
Average *B* factors (^2^)		
Protein		27.3
Solvent		35.0
Sulfate		33.4
Glycerol		39.6
DPI†† ()		0.138
Ramachandran plot		
Favoured (%)		97.1
Allowed (%)		2.9
Outliers (%)		0.0

† *R*_merge_ = 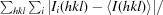 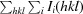, where *I_i_*(*hkl*) is the intensity of the *i*th measurement of reflection *hkl* and *I*(*hkl*) is the mean value of *I*_*i*_(*hkl*) for all *i* measurements.

‡ *R*_p.i.m._, the precision-indicating merging *R* factor, is *R*_merge_ adjusted bya factor of [1/(*N* 1)]^1/2^, where *N* is the number of times a given reflection is observed.

§ *R*_work_ = 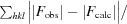 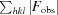, where *F*_obs_ is the observed structure-factor amplitude and the *F*_calc _ is the structure-factor amplitude calculated from the model.

¶ *R*_free_ is calculated with a subset of data that were excluded from refinement calculations (5%) using the same method as for *R*_merge_.

†† Diffraction-component precision index (Cruickshank, 1999 ▶).
